# Narrow-Band 311 nm Ultraviolet-B Radiation Evokes Different Antioxidant Responses from Broad-Band Ultraviolet

**DOI:** 10.3390/plants10081570

**Published:** 2021-07-30

**Authors:** Arnold Rácz, Éva Hideg

**Affiliations:** Department of Plant Biology, University of Pécs, Ifjúság útja 6, 7624 Pécs, Hungary; dlonra1991@gmail.com

**Keywords:** UV-B, hydrogen peroxide, peroxidase, superoxide dismutase, non-enzymatic antioxidants, acclimation

## Abstract

Supplemental narrow-band 311 nm UV-B radiation was applied in order to study the effect of this specific wavelength on tobacco as a model plant. UV-B at photon fluxes varying between 2.9 and 9.9 μmol m^−2^ s^−1^ was applied to supplement 150 μmol m^−2^ s^−1^ photosynthetically active radiation (PAR) for four hours in the middle of the light period for four days. Narrow-band UV-B increased leaf flavonoid and phenolic acid contents. In leaves exposed to 311 nm radiation, superoxide dismutase activity increased, but phenolic peroxidase activity decreased, and the changes were proportional to the UV flux. Ascorbate peroxidase activities were not significantly affected. Narrow-band UV-B caused a dose-dependent linear decrease in the quantum efficiency of photosystem II, up to approximately 10% loss. A parallel decrease in non-regulated non-photochemical quenching indicates potential electron transfer to oxygen in UV-treated leaves. In addition to a flux-dependent increase in the imbalance between enzymatic H_2_O_2_ production and neutralization, this resulted in an approximately 50% increase in leaf H_2_O_2_ content under 2.9–6 μmol m^−2^ s^−1^ UV-B. Leaf H_2_O_2_ decreased to control levels under higher UV-B fluxes due to the onset of increased non-enzymatic H_2_O_2_- and superoxide-neutralizing capacities, which were not observed under lower fluxes. These antioxidant responses to 311 nm UV-B were different from our previous findings in plants exposed to broad-band UV-B. The results suggest that signaling pathways activated by 311 nm radiation are distinct from those stimulated by other wavelengths and support the heterogeneous regulation of plant UV responses.

## 1. Introduction

**Figure 1:**
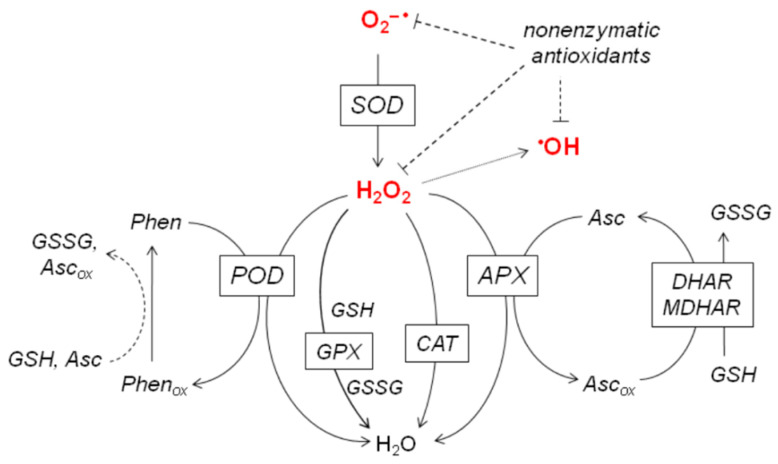
Major antioxidants participating in ROS neutralization. Solid and dashed lines represent enzymes and non-enzymatic reactions, respectively. Nonenzymatic antioxidants include ascorbate (Asc), glutathione (GSH), phenolic compounds (Phen), and several other compounds. Their reaction with ROS is symbolized with bar-ended dashed lines. Superoxide anion radicals O_2_^−•^ are converted to hydrogen peroxide (H_2_O_2_) by superoxide dismutase (SOD). A dotted arrow shows the possibility of the conversion of H_2_O_2_ into hydroxyl radicals (^•^OH), which is prevented by the decomposition of H_2_O_2_. H_2_O_2_ neutralizing enzymes include ascorbate peroxidase (APX), catalase (CAT), glutathione peroxidase (GSH), and class III peroxidases (POD). Oxidized ascorbate (Asc_ox_) is reduced by monodehydroascorbate reductase and dehydroascorbate reductase enzymes, which also produce glutathione disulfide (GSSG). Some oxidized phenolic compounds (Phen_ox_) can be recovered by Asc or GSH.

A key function of plant stress tolerance is the maintenance of relatively low cellular concentrations of reactive oxygen species (ROS). An overview of enzymatic and non-enzymatic antioxidant functions is shown in Figure 1. When a stressor stimulates existing cellular ROS-generating mechanisms or activates new pathways, oxidative damage can be avoided by re-adjusting ROS levels via changes in antioxidants, and these processes have been reviewed extensively [1,2,3,4,5]. Our previous experiments showed that it is possible to acclimate “UV naïve” plants to supplemental UV-B (280–315 nm) in a growth chamber without a loss of photochemical activity, even under higher than ambient ratios of UV to photosynthetically active radiation (PAR). Using a broad-band UV source centered at 313 nm, we previously showed that maintaining an effective H_2_O_2_-regulating system is essential to avoid or reduce the loss of photosynthetic function [6,7,8]. Plants respond to several potential stressors by enhancing peroxidase activity [9,10], but this line of defense is of special importance in response to UV-B because short-wave (λ < 305 nm) radiation is capable of photoconverting H_2_O_2_ to hydroxyl radicals [11]. In the above-cited studies, ascorbate peroxidase (APX) and class III peroxidase (POD) enzyme activities were 1.5–9 times more active in leaves acclimated to broad-band UV-B radiation than in controls. The extent of the increase depended on leaf age [8,12], the model species (*Arabidopsis thaliana*, *Nicotiana benthamiana*, or *Nicotiana tabacum*), and the applied biologically effective daily dose (between 3.2 and 7.7 kJ m^−2^ d^−1^, plant weighted according to Flint and Caldwell [13]). Experiments with *N. tabacum* suggested a phenolic substrate-dependent heterogeneity in the POD response and identified chlorogenic acid (CGA), a dominant secondary metabolite in tobacco [14], as the preferred substrate of the most UV-responsive POD isoforms [15].

The molecular regulation of the observed changes in POD enzymes in UV-B-irradiated leaves is still unknown. A large number of plant responses are regulated by UV RESISTANCE LOCUS 8 (UVR8), the UV-B photoreceptor [16], which is most sensitive to short-wavelength (290–300 nm) UV-B [17,18]. The photoreceptor protein forms a homodimer in the absence of UV-B, and its UV-B-induced monomerization and subsequent interaction with the CONSTITUTIVELYPHOTOMORPHOGENIC 1 (COP1) protein initiates signal transduction and transcription of target genes involved in UV-B responses [16,17,18,19,20], such as DNA repair, protection of photosynthetic machinery and production of UVB-absorbing phenylpropanoids [21,22]. It is important to note that not all UV-B-inducible genes are UVR8-regulated and that UVR8 has a broader effect on plant growth and development beyond mediating UV-B responses. Only one UV-B-induced and UVR8-regulated peroxidase gene has been identified in response to UV-B: a glutathione peroxidase (GPX) in *A. thaliana* [23]. Another gene was found to respond specifically to UV-B stress in the same species [24], but it was subsequently identified as a cell-wall-bound class III peroxidase associated with growth limitation [25] rather than an enzyme with antioxidant function. Some UVR8-independent UV-B responses have been proposed to be ROS-mediated, especially under higher UV fluence rates [24]. Hydrogen peroxide has been suggested to be part of the signaling pathway in a variety of stress conditions [2,4,26,27] and is a plausible alternative to the photoreceptor-mediated mechanism because UV treatment increases leaf H_2_O_2_ levels. However, we recently found that increasing leaf content by directly treating *N. tabacum* plants with exogeneous H_2_O_2_ did not increase leaf POD activities and induced antioxidant responses that were distinct from those elicited by UV-B irradiation [15]. Therefore, it appears that POD responses to UV-B involve either a metabolic signal other than H_2_O_2_ or a photoreceptor other than UVR8. To investigate the latter possibility, we used a monochromatic UV-B source that emits outside the known absorption range of UVR8 [18,28].

Recently, a UVR8- and stress-independent UV-B signaling pathway (UASI) was proposed to operate under low, non-damaging doses of lower-energy UV-B with a maximum response at around 310 nm [29]. In the present study, we used the same narrow-band UV-B source as the authors of the above study in order to investigate whether this UV-B wavelength has specific effects on leaf H_2_O_2_ levels and related antioxidant responses. Because our experience has shown that tobacco plants are more UV-B tolerant than Arabidopsis, the UV-B fluxes applied in the present study (3–9 μmol m^−2^ s^−1^) were higher than those in the study by O’Hara et al. [29] (0.1–3 μmol m^−2^ s^−1^).

## 2. Results

**Figure 2:**
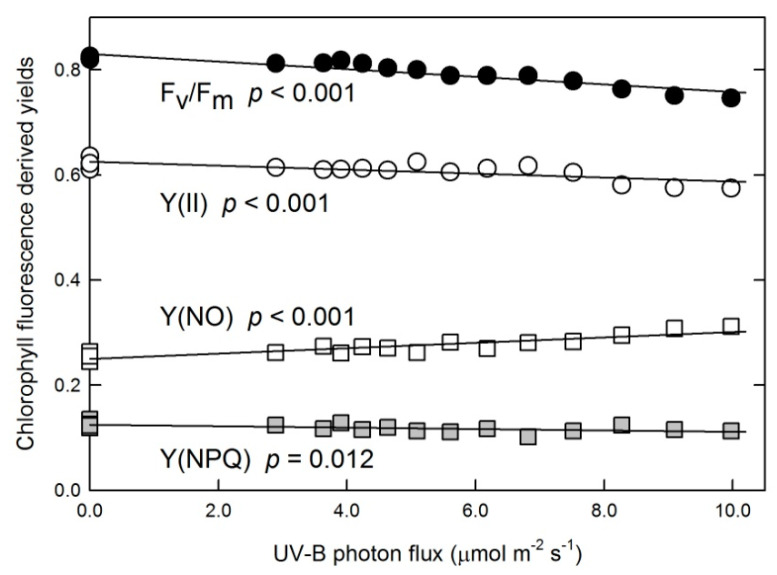
Changes in photochemical yields F_v_/F_m_ and Y(II), regulated non-photochemical quenching Y(NPQ), and non-regulated non-photochemical quenching Y(NO) in tobacco leaves exposed to various UV-B photon fluxes. Symbols represent data of individual leaves and solid lines show linear fits characterized by corresponding *p*-values.

The experimental setup allowed us to perform a gradient experiment, exposing each plant to different UV-B conditions (see Section 4.1.) while applying the same PAR, temperature, and humidity to all plants. Figure 2 shows that both photochemical and non-photochemical yields were affected by 311 nm UV-B. As UV-B flux increased, light-acclimated Photosystem II quantum efficiency and regulated non-photochemical quenching (Y(II) and Y(NPQ), respectively) decreased, while non-regulated non-photochemical quenching Y(NO) increased. The relative change was the largest in Y(NO), which increased by 20% in response to the highest UV-B flux, while Y(II) and Y(NPQ) decreased by 6% and 11%, respectively. The maximum quantum efficiency of Photosystem II (F_v_/F_m_) also decreased linearly as UV-B increased, with a 9% decrease under the strongest treatment.

**Figure 3:**
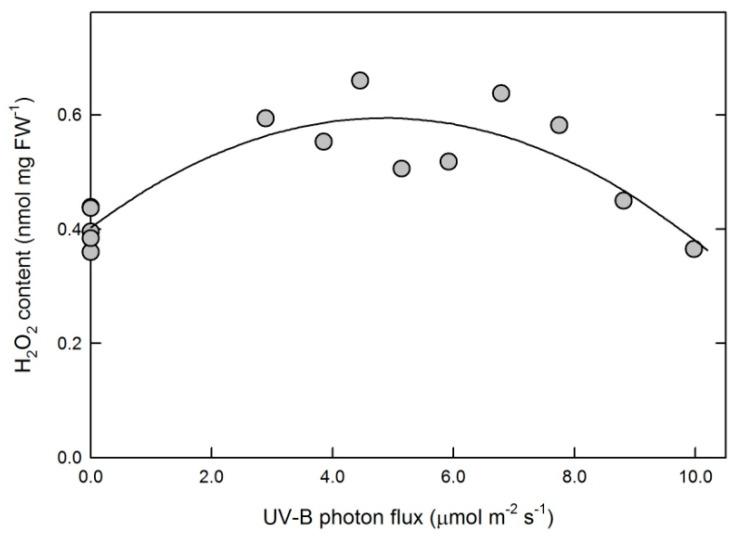
Changes in leaf H_2_O_2_ contents in tobacco leaves exposed to various UV-B photon fluxes. Symbols represent data of individual leaves and the solid line shows the result of a second order polynomial fit characterized by *p* < 0.001.

Leaf H_2_O_2_ contents did not follow a linear trend as a function of 311 nm UV-B flux, and the observed rise-and-fall kinetics were well approximated by a second-order polynomial regression model (*p* < 0.001) (Figure 3).

**Figure 4:**
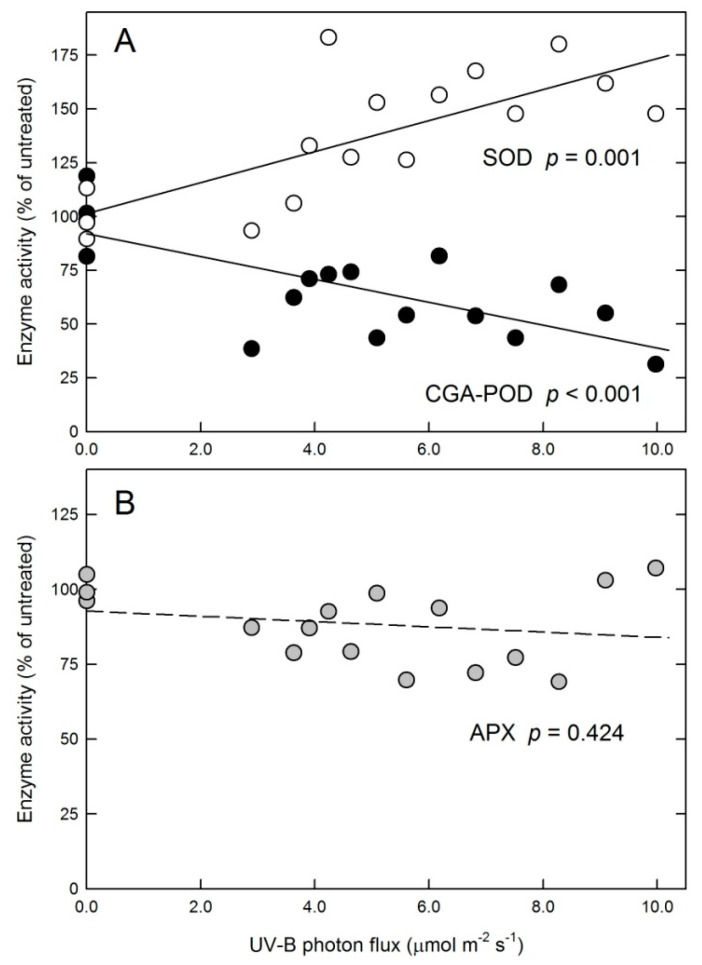
Changes in antioxidant enzyme activities. (**A**) Superoxide dismutase (SOD) and peroxidase enzyme activity assayed with chlorogenic acid (CGA-POD). (**B**) Ascorbate-peroxidase enzyme activity (APX).

Antioxidant enzymes affecting H_2_O_2_ content were also measured in each plant, and the results are shown in Figure 4. Superoxide dismutase (SOD) enzyme activities increased as UV-B increased, and a statistically significant linear model described the observed trend (Figure 4A). Peroxidase (POD) activity was assayed using chlorogenic acid (CGA). As previously reported, this method results in higher activities in tobacco grown as a model than the use of other electron donors, such as ABTS or guaiacol [15]. POD activity measured with CGA (CGA-POD) followed a linearly decreasing trend as 311 nm flux increased (Figure 4A). Ascorbate peroxidase (APX) activities were less affected by UV-B than either SOD or CGA-POD (Figure 4B), and neither the linear nor the polynomial regression analysis revealed significant dependence on UV-B fluxes (data not shown). Because the extent of the POD activity response to broad-band UV-B was found to depend on the choice of substrate, POD was also assayed in this study with ABTS. However, regression analysis of ABTS-POD indicated that neither the linear nor polynomial trend were significant (data not shown).

**Figure 5:**
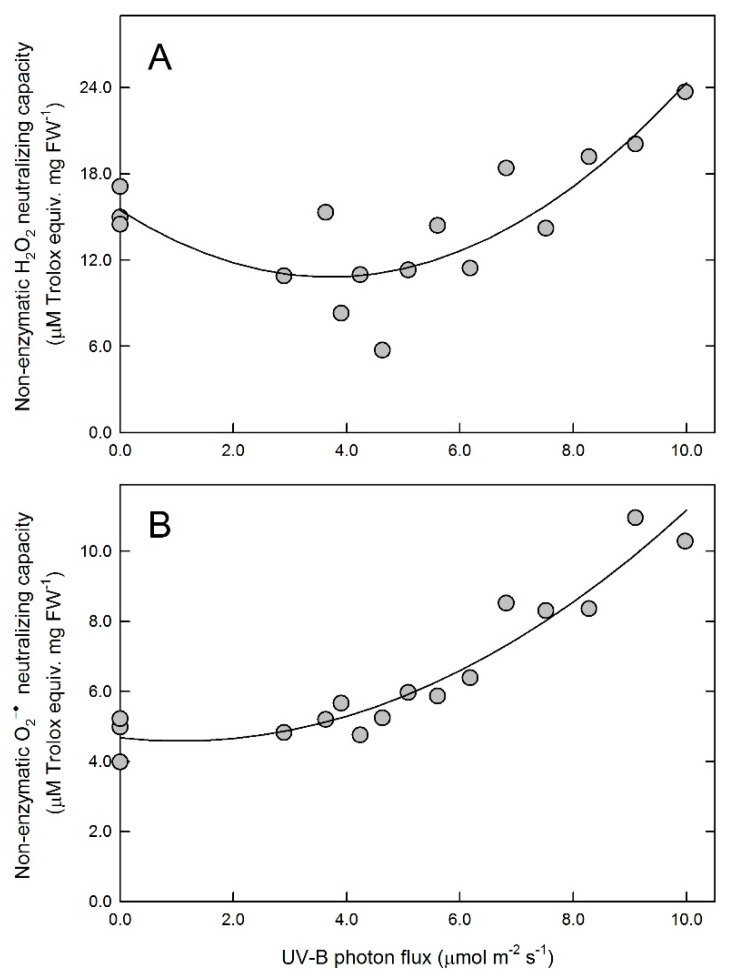
Changes in non-enzymatic hydrogen peroxide (**A**) and superoxide anion radical neutralizing (**B**) capacities. Symbols represent data of individual leaves and lines show the results of a second order polynomial fit characterized by *p* < 0.001 for both data sets.

Non-enzymatic H_2_O_2_- and O_2_^−•^-neutralizing capacities were measured using ethanolic leaf extracts, which had no enzymatic antioxidant activities. When the results were examined as a function of the applied UV-B flux, changes in H_2_O_2_ scavenging were different from those in O_2_^−•^ neutralization, and neither parameter followed a linear trend (Figure 5). Non-enzymatic ROS-neutralizing capacities of leaves treated with narrow-band UV-B at low flux rates were slightly different from those of untreated leaves, but the differences were more pronounced in samples exposed to higher fluxes. UV-induced changes in non-enzymatic H_2_O_2_ antioxidants were smaller, and the trend suggests a transient decrease before a small increase in response to the highest applied fluxes (Figure 5A). Non-enzymatic O_2_^−•^-neutralizing capacities followed a gradually increasing trend, and there was an approximately two-fold difference between untreated and high-flux-exposed samples (Figure 5B).

**Figure 6:**
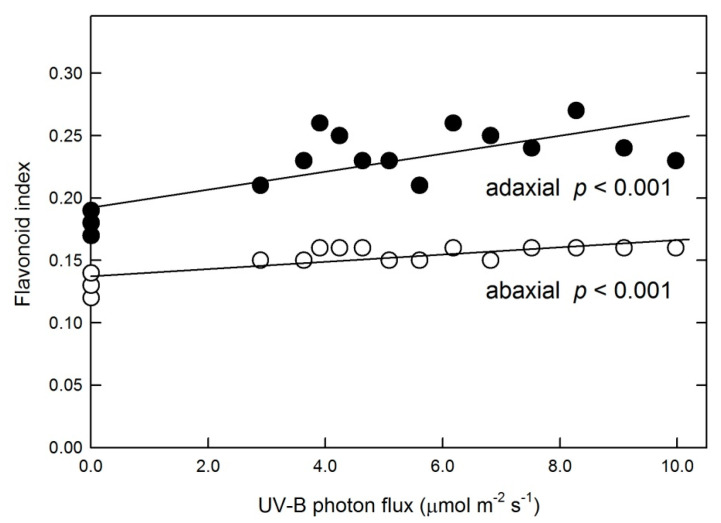
Changes in adaxial (filled symbols) and abaxial (empty symbols) flavonoid index values in response to various UV-B photon fluxes. Symbols represent data of individual leaves and lines show results of linear fits characterized by corresponding *p*-values.

Acclimation responses to broad-band UV-B include increased biosynthesis of phenolic products; therefore, these compounds were also measured in plants exposed to the narrow-band UV-B. Irradiation with 311 nm UV-B resulted in an increase in the Dualex flavonoid index, which characterizes the epidermal flavonoid content by measuring leaf absorbance at 375 nm. This parameter showed a linearly increasing trend on both adaxial and abaxial leaf sides (Figure 6). Thin-layer chromatography confirmed increased flavonoid levels in UV-B-treated leaves and also indicated higher amounts of phenolic acids (Supplementary Figure S2).

**Figure 7:**
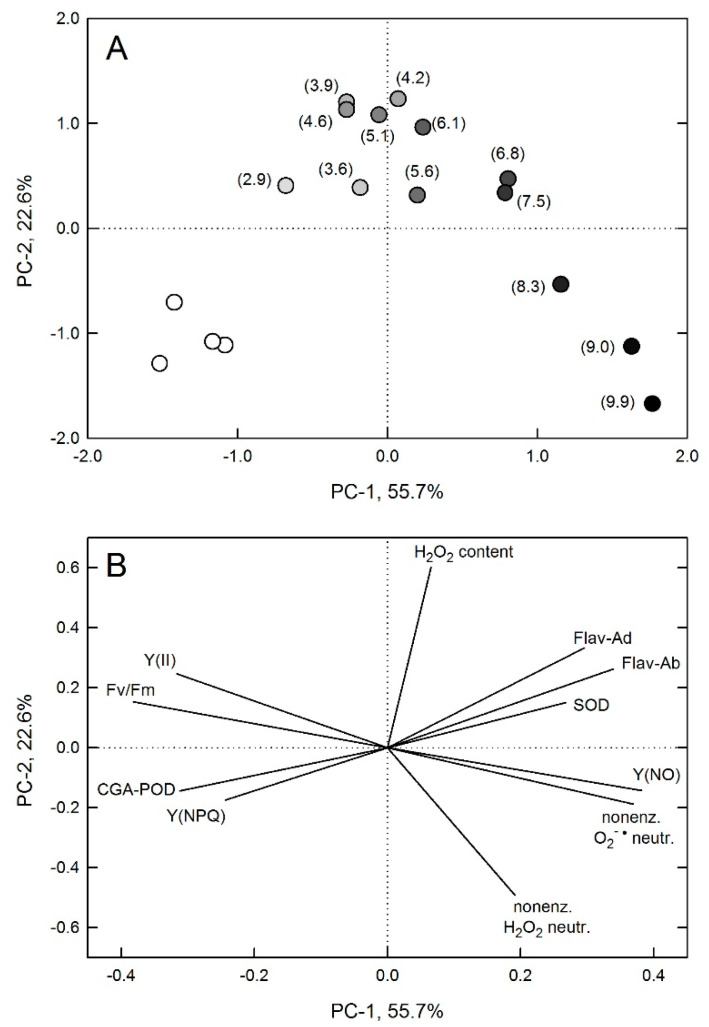
Principal component (PC) analysis of correlations. (**A**) Data points. Empty symbols mark untreated leaves and filled symbols represent UV-B treated leaves: darker shades of grey corresponding to higher fluxes. Numbers in parenthesis are UV-B fluxes in μmol m^−2^ s^−1^ units. (**B**) Projections of UV-B responsive original variables. Adaxial and abaxial leaf flavonoid index (Flav-Ad and Flav-Ab, respectively); non-enzymatic hydrogen peroxide and superoxide neutralising capacities (nonenz. H_2_O_2_ neutr. and nonenz. O_2_^−•^ neutr., respectively); maximum and light acclimated photochemical quantum yields (F_v_/F_m_ and Y(II), respectively); yields of regulated and non-regulated non-photochemical quenching (Y(NPQ) and Y(NO), respectively); superoxide dismutase (SOD) and chlorogenic acid dependent peroxidase (CGA-POD) enzyme activities; and leaf H_2_O_2_ content.

Correlations of leaf photochemical yields, H_2_O_2_ content, and various potentially protective responses were also analyzed using PCA. Data from plants in UV-B-free conditions and those treated with 311 nm were clustered, and those treated with higher and lower UV-B fluxes were separated (Figure 7A). The projection of leaf H_2_O_2_ content was separated from all other parameters, similar to data from leaves under 4–6 μmol m^−2^ s^−1^ 311 nm photon flux densities. SOD and CGA-POD activities were opposite to each other, corresponding to reverse trends in their changes. Photochemical yields F_v_/F_m_ and Y(II) were strongly negatively correlated with Y(NO), which was in the same quadrant as non-enzymatic antioxidant capacities and data from leaves under higher UV-B fluxes. Projections of epidermal flavonoid contents were separated from non-enzymatic antioxidant capacities (Figure 7B).

## 3. Discussion

Acclimation to UV-B includes a combination of diverse signaling and protective and repair mechanisms. The exact coordination of these processes with other environmental factors in plants growing under natural sunlight, where UV-B alone is usually not damaging [30], is yet to be explored. Model experiments, on the other hand, provide the possibility of separating factors. Responses to single low doses of UV-B (such as 0.1–1 μmol m^−2^ s^−1^ photon flux applied for 15–60 min) are initiated by the UVR8 photoreceptor [19]. On the other hand, the existence of UVR8-independent regulation has also been recognized and has been suggested to be triggered by longer-wavelength UV-B [29,31,32]. Because UV-B exposure results in considerable alterations in antioxidant status, the role of ROS as alternative signals has also been proposed [33,34,35,36]. As we showed earlier, UV tipped the balance of enzymatic H_2_O_2_ production and neutralization in favor of the latter by markedly increasing peroxidase activities. Depending on the applied UV-B dose and the studied species, H_2_O_2_-yielding SOD activity (i) also increased, but to a lesser extent than peroxidase activities [6,7,37], (ii) which showed no marked change [38] or (iii) which decreased [15] in UV-treated plants as compared to controls. However, to date, the role of low-energy UV-B in this process has not been explored. Our experiments with quasi-monochromatic 311 nm UV-B show that when this treatment is applied alone, the changes in H_2_O_2_ regulation differ from those resulting from a broad-band UV source containing a range of UV wavelengths in addition to 311 nm.

There is an important yet indefinite distinction between stressing and non-stressing UV treatments. Conditions resulting in detectable levels of ROS are sometimes regarded as stressing. Gene expression studies usually apply Arabidopsis plants under single low doses of UV-B (such as 0.1–1 μmol m^−2^ s^−1^ photon flux applied for 15–60 min) in order to avoid overlapping with such stress effects. Our approach applies the definition that considers plants to be UV-B-stressed when they are unable to tolerate UV-B levels and manage the resulting cellular damage [36]. Tobacco plants in growth chamber experiments have been found to acclimate to higher UV-B fluxes without a major loss of photochemical function [15]. From a photobiological point of view, it is impossible to compare the effects of two UV-B sources unless their emission spectra are identical [39]. However, a study on the effect of separate UV-B energies requires compromises. Therefore, we applied a range of 311 nm UV-B photon fluxes, starting from about 60% of the 311.5 ± 2.5 nm flux contained in the broad-band source applied in the above experiment (Supplementary Figure S1A). Although an extension of this flux range might be desirable, this is prevented by technical constraints, such as the undimmable nature of the applied UV-B source and the maximum distance between the source and plant inside the chamber.

### 3.1. Photosystem II Is Limited by Long-Wavelength UV-B and Is a Potential Source of ROS

Trend analyses of data from plants exposed to gradually increasing 311 nm UV-B radiation fluxes show a small but gradual decline in photochemical yield (Figure 2). Projections of PCA show that F_v_/F_m_ and the light-acclimated yield Y(II) changed in parallel, which indicates a direct UV-B effect on PS II rather than a downregulation via regulated non-photochemical quenching Y(NPQ). This is supported by the strong negative correlation between photochemical and non-regulated non-photochemical quenching yields (Y(II) and Y(NO), respectively) (Figure 2 and Figure 6). Y(NO) is the result of two combined pathways: the first part consists of intrinsic (light-independent) non-radiative de-excitation reactions, and the second part of this quenching is caused by photoinhibition or other processes including triplet states [40,41]. In leaves exposed to 311 nm UV-B, the activities of both pathways increased. An increase in non-radiative de-excitation in UV-B-treated leaves is indicated by decreasing F_v_/F_m_ because, in the dark-adapted state, Y(NO) = 1-F_v_/F_m_ [41]. The gradual increase in the second, light-dependent part of Y(NO) in response to increasing UV-B fluxes is demonstrated by the linear decrease in both Y(II) and Y(NPQ) because, in the PAR-acclimated state, Y(NO) = 1-Y(II)−Y(NPQ) [41]. The light-dependent part of Y(NO) contributes to lower Y(NPQ)/Y(NO) in 311 nm–treated leaves as compared to non-UV controls, which reflects a suboptimal capacity of photoprotective reactions and the possibility of ROS production via either energy or electron transfer to oxygen. It is noted here that the maximum extent of photochemical yield losses estimated from the observed trends is relatively low (less than 10%) (Figure 2). Therefore, the amount of singlet oxygen from the first reaction, if any, is not expected to be directly detectable. In an earlier experiment, spinach leaves were exposed to a much stronger UV-B treatment (310 ± 5 nm UV-B for 15 min at 35 μmol m^−2^ s^−1^ photon flux), and although they lost 30% of their photochemical yields, very little singlet oxygen was detected in these samples [42]. ROS from the second reaction type, the superoxide anion radical, may be neutralized on site via SOD and APX in the water–water cycle (Asada–Halliwell cycle) of the chloroplast stroma [5,43]. If the activities of enzymes participating in this pathway do not increase in response to higher ROS yields, superoxide-derived H_2_O_2_ may leave the chloroplast [44,45], where its local concentrations are controlled by peroxidases.

### 3.2. Long-Wavelength UV-B Tips the Balance between Enzymatic H_2_O_2_ Production and Scavenging

Leaf H_2_O_2_ levels were affected by the applied 311 nm treatment. A rise-and-fall trend was observed and approximated by a second-order polynomial fit (Figure 3). More complex mathematical models may also apply but were not attempted. Due to the possibility of H_2_O_2_ diffusion between cellular compartments, the primary source of this ROS can only be speculated. The above-discussed UV-B-induced changes in leaf photochemistry and non-photochemical quenching imply a possible plastid origin. Although the applied activity assays performed on whole-leaf extracts do not allow for a distinction between responses of plastid and non-plastid enzymes to 311 nm UV-B, the lack of a significant APX response indicates that full activation of the water–water cycle is unlikely. Assuming that the observed total SOD activities include chloroplast SOD, a selective increase in SOD without a matching increase in APX activity would increase plastid H_2_O_2_ levels. Surprisingly, the response of these two enzymes to 311 nm UV-B (increasing SOD and unchanged APX, Figure 4) was opposite to previous observations in leaves acclimated to broad-band UV-B (decreasing or unchanged SOD and increasing APX [7,8,15,38]). This discrepancy may be explained by the potential photocleavage of H_2_O_2_ to hydroxyl radicals by shorter (285–305 nm), but not longer, UV-B wavelengths [11], leading to the stricter control of cellular H_2_O_2_ levels in the presence of shorter-wavelength UV-B than under 311 nm UV-B only.

### 3.3. Non-Enzymatic Antioxidants Respond to Higher Fluxes of Long-Wavelength UV-B Than Enzymes

Although SOD activity linearly increased and POD activity decreased with increasing 311 nm fluxes (Figure 4A), this did not result in a monotonous rise in leaf H_2_O_2_ levels (Figure 3). An increase in H_2_O_2_ concentrations under lower UV-B flux rates as compared to control plants is explained by the responses of these two key enzymes to the treatment. A decline under higher flux rates, however, is due to the increase in the removal of H_2_O_2_ and especially O_2_^−•^ by non-enzymatic antioxidant mechanisms, which was not detectable in leaves exposed to lower doses. As we reported previously, broad-band UV-B or treatment with exogenous H_2_O_2_ both increased leaf H_2_O_2_ contents by 30–50%, but only the latter stimulated non-enzymatic ROS scavenging [15]. Therefore, it is less likely that increased ROS concentrations triggered the non-enzymatic antioxidant response in the present study. A more plausible explanation is an antioxidant response to the negative effect of higher (>7.5 μmol m^−2^ s^−1^) 311 nm fluxes on leaf photochemistry, which was observed both in the above-cited study using broad-band UV-B and under lower 311 nm fluxes in this experiment. In addition to causing a 25% decrease in photochemical yields, exposure of Arabidopsis leaves to broad-band UV-B also produced an increase in non-enzymatic antioxidant capacities [38]; therefore, the positive effect observed in this study (Figure 6) is a consequence of high UV photon flux rather than a unique characteristic of 311 nm UV-B, as opposed to the effect of 311 nm treatment on antioxidant enzymes (Figure 4), which appear to be specifically driven by this wavelength. The chemical nature of antioxidants that cause such an increase is yet to be explored, but the contribution of flavonoids appears to be minor, as shown by the distinct responses of leaf flavonoids and non-enzymatic antioxidant capacities (Figure 5 and Figure 6).

In summary, our results suggest that broad-band UV sources that include an array of wavelengths and distinct energy ranges may contribute to different response mechanisms in plants. Due to technical limitations, comparative studies on the effects of distinct UV wavelengths or narrow UV wavelength ranges have not been realized thus far, although they would contribute to our understanding of responses to the whole UV spectrum both in model experiments and outdoors. The usual method is the application of a series of UV cut-off filters, although this approach may change with the improvement of UV LED technology. The present work suggests that 311 nm UV-B and other wavelengths contained in the broad-band source have opposite effects on antioxidant enzymes, but the effect of long-wavelength UV-B is overcome by the composite effect of other wavelengths. The presented unique 311 nm responses support the involvement of a putative UV-B photoreceptor distinct from UVR8 [29], but the presented data only represent the first steps of further research on this topic. As a potential application in horticulture, treatment with moderate 311 nm fluxes for a few days may be utilized to achieve a temporal increase in leaf H_2_O_2_ levels in plants grown indoors without the risk of photoconversion into damaging hydroxyl radicals. Such pre-treatment would prepare plants for subsequent stress, such as transport or changes in light, humidity, and soil conditions during agricultural practice. Based on the present study, fluxes in the 3–6 μmol m^−2^ s^−1^ range may be suitable for this purpose, but this is expected to depend on plant species, age, and growth conditions.

## 4. Materials and Methods

### 4.1. Plant Material and Treatments

Tobacco (*Nicotiana tabacum* cv. Petit Havana) plants were grown on garden soils in 8 × 8 cm rectangular plastic pots in a growth chamber (Fitotron, SGC 120 Plant Growth Chamber, Weiss Technik UK, Loughborough, UK) under 150 μmol m^−2^ s^−1^ photosynthetically active radiation (PAR) using long-day conditions (16/8 h, 25/20 °C) and 65% relative humidity. These conditions were maintained throughout the 4-day UV treatment period, during which PAR was supplemented with narrow-band UV-B radiation centered at 311 nm for 4 h daily. For this treatment, 5-week-old plants were aligned side by side under a Philips TL20 W/01RS tube (Philips Electronics, Roosendaal, The Netherlands), which was mounted at an angle inside the chamber in order to provide various photon flux densities for the experiment. UV responses of the last fully developed leaf were evaluated in the experiment (one leaf per plant), and the vertical distance between the leaf and the narrow-band source varied between 5 and 52 cm. In order to remove shorter-wavelength UV components, the tube was wrapped in a single layer of cellulose diacetate filter (Courtaulds Chemicals, Derby, UK). Control plants were kept in the same growth chamber but were shielded from the UV-B source with a UV-excluding plastic foil (Rosco 3114 UV filter, Roscolab Ltd. London, UK) during the four treatment days.

Spectral irradiance was measured with a spectroradiometer (Flame, Ocean Optics, Largo, FL, USA), and it was established that UV-B irradiation was centered at 311.5 nm with 2.5 nm full width at half-maximum (Supplementary Figure S1A). Therefore, quasi-monochromatic radiation from this source is referred to as 311 nm UV-B in the present study. Depending on the distance from the source, leaves were exposed to 2.9–9.9 μmol m^−2^ s^−1^ 311 nm photon flux densities (Supplementary Figure S1B). The 4-h exposure corresponded to 4.6–16 kJ m^−2^ d^−1^ biologically effective UV-B calculated using the biological spectral weighting function developed for plants [13] and the emission spectrum of the source. These doses are comparable to the 6.9 kJ m^−2^ d^−1^ biologically effective dose applied in our previous experiments using a broad-band UV source [8] (Q-Panel UVB-313EL tubes, Q-Lab Ltd., Bolton, UK) (see Supplementary Figure S1A for emission spectra).

At the end of the narrow-band UV-B irradiation period on the 4th day of the treatment, non-invasive measurements were performed to assess pigment content and chlorophyll fluorescence-derived parameters. The last fully developed leaf was used from each plant in all experiments, and it was detached after completing the above non-invasive measurements. After measuring fresh weights, leaves were frozen in liquid N_2_ and stored at −60 °C for further analyses. In a separate experiment repeated under the same irradiation conditions, leaf H_2_O_2_ contents were measured from leaf disks cut in situ while plants were inside the growth chamber.

### 4.2. Chlorophyll Fluorescence Measurements

Plants were kept in darkness for 30 min before photochemical yields and non-photochemical quenching were characterized by chlorophyll fluorescence-derived parameters using the MAXI-version of the Imaging PAM (Heinz Walz GmbH, Effeltrich, Germany). At the end of the dark adaptation period, a saturating pulse was applied in order to measure the minimum and maximum fluorescence yields (F_0_: before the pulse; F_m_: after the pulse). The maximum Photosystem (PS) II quantum yield was calculated from these data [46] as F_v_/F_m_ = (F_m_−F_0_)/F_m_. Following this, the plant was illuminated with blue actinic light corresponding to 110 μmol m^−2^ s^−1^ PAR for 5 min, and then the F′ and F′_m_ fluorescence yields were measured before and after a saturating pulse. The light-acclimated, effective PS II quantum yield was calculated from these data as Y(II) = (F′_m_−F′)/F′_m_, and the yields of non-regulated and regulated non-photochemical quenching were characterized by Y(NO) = F’/F_m_ and Y(NPQ) = F′/F′_m_−F′/F_m_, respectively [41].

### 4.3. Leaf Flavonoid Contents

Adaxial and abaxial side flavonoid contents were estimated using a non-invasive optical method using the Dualex Scientific™ optical sensor [47] (ForceA, Orsay, France). In addition, phenolic compounds were analytically separated by thin-layer chromatography (TLC) performed on silica gel-coated aluminum sheets (60 F_254_, 12 × 20 cm; Merck KGaA, Darmstadt, Germany). The test compounds chlorogenic acid and quercetin-3-*O*-glucoside were purchased from Sigma-Aldrich Kft (Budapest, Hungary) and Extrasynthese S.A.S. (Genay, Rhone, France), respectively. In order to visualize major UV-responsive phenolic compounds, leaf extracts (0.3 mg leaf FW mL^−1^ in ethanol) and test compounds (1 mg mL^−1^ in ethanol) were applied on the TLC plate at 5 μL volumes. The plate was developed in a mixture of ethyl acetate, formic acid, acetic acid, and water; phenolic compounds were visualized under UV light as described previously [15].

### 4.4. Enzyme Activity Measurements

Frozen leaves were powdered in liquid N_2_ using a pestle and mortar and extracted in an ice-cold sodium-phosphate buffer (50 mM, pH 7.0) containing 1 mM EDTA. Leaf homogenates were centrifuged (24,400× *g* for 30 min at 4 °C, Hettich Rotina 380 R, Andreas Hettich GmbH, Tuttlingen, Germany), and the supernatants were used for all the antioxidant enzyme assays. When making samples for the ascorbate peroxidase assay, 5 mM ascorbate was also included in the grinding buffer.

Class III peroxidase (POD, EC 1.11.1.7) activities were measured in acidic reaction mixtures (50 mM phosphate citrate buffer, pH 5.0) using either 2,2′-azino-bis-(3-ethylbenzothiazoline-6-sulfonic acid (ABTS) or chlorogenic acid (CGA) [15]. Enzyme activities were quantified following the oxidation of the corresponding substrate at either 756 nm (ABTS) or 325 nm (CGA) using a spectrophotometer (Shimadzu UV1800, Shimadzu Corp., Kyoto, Japan). Activities were calculated using molar extinction coefficients of the corresponding substrates and expressed in U POD mg^−1^ leaf FW.

Superoxide dismutase (SOD, EC 1.15.1.1) activity assays were carried out according to Sun et al. [48]. Xanthine and xanthine oxidase (2 mM and 25 mU, respectively) were used as a superoxide anion source, and the conversion of nitroblue tetrazolium (NBT, 0.1 mM) to formazan was detected at 560 nm with a spectrophotometer. Results are reported as U SOD mg^−1^ leaf FW.

Ascorbate peroxidase (APX, EC 1.11.1.11) activity was measured by following the oxidation of ascorbate at 295 nm with the spectrophotometer in a sodium phosphate buffer (50 mM, pH 7.0) containing 1 mM EDTA, 0.5 mM ascorbate, and 1 mM H_2_O_2_ [49]. The results were corrected for APX-independent H_2_O_2_ reduction, which was typically less than 10% of enzymatic rates. Enzyme activities were calculated as mU APX mg^−1^ FW.

### 4.5. Non-Enzymatic Antioxidant Capacity Measurements

Aliquots of frozen leaf powder (150 mg) were mixed with 0.5 mL 70:30 (*V*/*V*) ethanol/water, incubated for 24 h at 4 °C in darkness, and then centrifuged (10,000× *g* for 10 min at room temperature). Supernatants were used to evaluate non-enzymatic ROS-neutralizing capacities using absorbance data collected with a Multiskan FC plate reader (Thermo Fisher Scientific, Shanghai, China).

Hydrogen peroxide-neutralizing antioxidant capacities were assessed via the photometric detection of iodine produced by the oxidation of potassium iodide by H_2_O_2_ and the ability of leaf extracts to lessen the amount of this product. Each 200 μL reaction mixture was mixed in a well of a microplate and contained 25 μM H_2_O_2_ and 15–20 μL leaf extract in 50 mM potassium-phosphate buffer (pH 7.0) [50]. Absorption at 405 nm was measured immediately after mixing the assay components and then after 2, 4, and 6 min. The increase in absorption was compared to the results of calibration measurements using 0.42–3.2 mM Trolox in order to obtain leaf results as μM Trolox equivalents mg^−1^ FW.

The superoxide radical (O_2_^−•^)-neutralizing antioxidant capacities were measured based on the capacities of the leaf extracts to inhibit the conversion of NBT to formazan. The 200 μL reaction mixture contained 0.3 mM xanthine, 0.3 mM EDTA, and 5–15 μL of leaf extract in 50 mM K-phosphate buffer (pH 7.2), and the reaction was started by adding 15 mU xanthine oxidase. Formazan production was measured as the increase in 540 nm absorbance, and the results were expressed as μM Trolox equivalents mg^−1^ FW.

### 4.6. Leaf Hydrogen Peroxide Content Measurement

Leaf H_2_O_2_ contents were estimated on the basis of the H_2_O_2_-induced absorption change in 125 μM xylenol orange in 6% (*v*/*v*) trichloroacetic acid (TCA), as described previously [15]. In order to avoid undesirable rapid changes in steady-state leaf H_2_O_2_ levels, leaf disks for this measurement were cut while plants were still in the growth chamber under light conditions corresponding to the treatment: PAR only or PAR plus UV-B. Three leaf disks corresponding to 26–56 mg FW were homogenized in 6% TCA immediately after cutting and then centrifuged (15,000× *g*, 10 min, 4 °C, Heraeus Fresco 17 Centrifuge, Thermo Fisher Scientific, Waltham, USA), and the supernatants were incubated for 30 min before detecting 560 nm absorptions. Leaf H_2_O_2_ contents were calculated in μmol g^−1^ FW units using calibration in the 0–10 nM H_2_O_2_ range.

### 4.7. Statistical Analysis

The UV experiment was carried out in a gradient design, and only non-UV controls were replicated (4–5 plants). Treatments with different UV irradiation fluxes were represented by one plant each (9–13 plants). Data (PAR-only controls and PAR + UV treatments) were analyzed by a gradient model as opposed to a replication model, and the effect of UV was characterized by regression analysis using UV-B photon flux as the independent variable and a biological parameter as the dependent variable (n = 14–18). With one exception, the leaf H_2_O_2_ content, all the UV-B-responsive biological parameters followed a linear trend. A UV-B-dependent increase or decrease in the observed parameter was regarded as significant when the slope of the fitting line was different from zero at *p* < 0.05, and residuals were normally distributed. Polynomial fits were only attempted when a linear regression model showed no significant association. Correlation and principal component analysis (PCA) were carried out in order to identify possible relationships between changes in the measured biological parameters. All statistical calculations were performed using the PAST software [51].

## Data Availability

Data is contained within the article.

## Supplementary Materials

The following are available online at https://www.mdpi.com/article/10.3390/plants10081570/s1. Figure S1A. Spectral characteristics of the applied narrow band UV-B tube in comparison with a broad band source. Figure S1B. Dependence of 311 nm UV-B flux on distance from the UV-B source. Figure S2. Thin layer chromatography of leaf extracts from 311 nm treated plants.

## Abbreviations

ABTS: 2,2′-azino-bis-(3-ethylbenzothiazoline-6-sulphonic acid); ABTS-POD: peroxidase EC 1.11.1.7 enzyme activity measured with ABTS as substrate; APX: ascorbate peroxidase EC 1.11.1.11 enzyme; CGA: chlorogenic acid; CGA-POD: peroxidase EC 1.11.1.7 enzyme activity measured with CGA as substrate; F_v_/F_m_: maximum quantum efficiency of PS II; PAR: photosynthetically active radiation; ROS: reactive oxygen species; RUT: quercetin-3-*O*-rutinoside; SOD: superoxide dismutase EC 1.15.1.1 enzyme; TLC: thin-layer chromatography; Y(II): quantum efficiency of light acclimated PS II; Y(NO): non-regulated non-photochemical quenching of PS II; Y(NPQ): regulated non-photochemical quenching of PS II.
