# Supplementary material for: Narrow-Band 311 nm Ultraviolet-B Radiation Evokes Different Antioxidant Responses from Broad-Band Ultraviolet
Source: Plants (Basel). 2021 Jul 30;10(8):1570. doi: 10.3390/plants10081570 (PMC8400681; doi:10.3390/plants10081570)
Supplement: Supplementary file 1 [file plants-10-01570-s001.zip › plants-1299367-SI.pdf]

Narrow-Band 311 nm Ultraviolet-B Radiation Evokes Different Antioxidant Responses from Broad-Band Ultraviolet

Arnold Rácz and Éva Hideg

Department of Plant Biology, University of Pécs, Hungary

Supplementary Materials

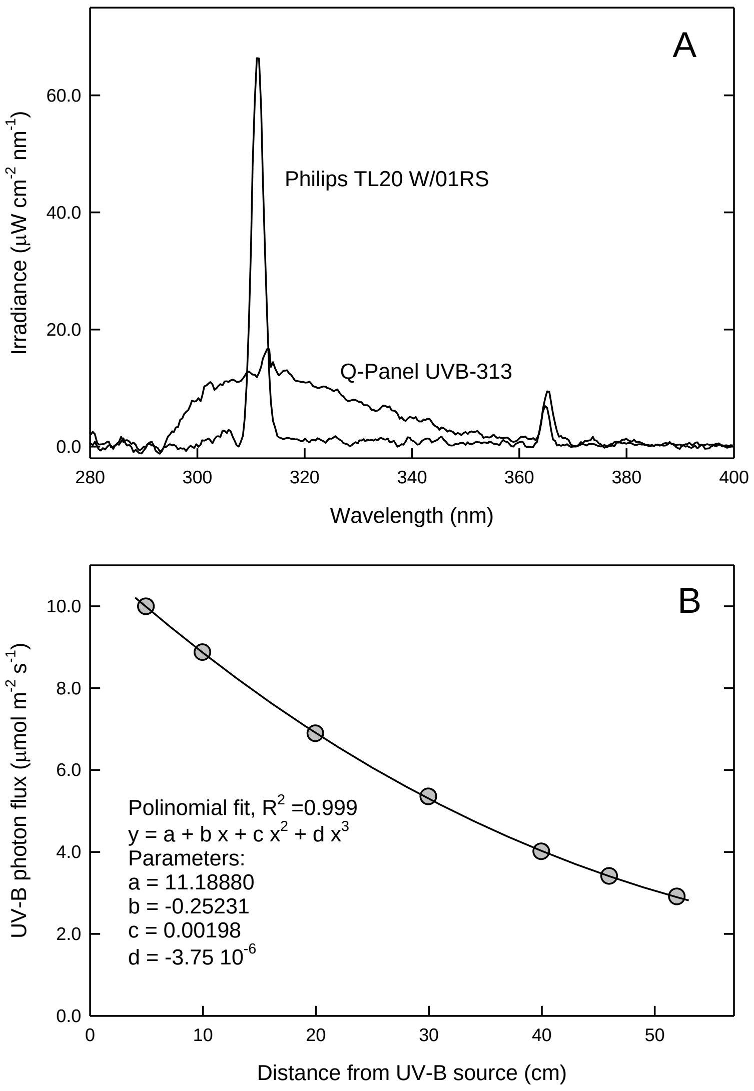

Supplementary Figure S1.

(A) Spectral characteristics of the applied narrow band UV-B tube applied in the present study in comparison with the broad band source applied earlier works mentioned here (Rácz et al. 2020). (B) Dependence of 311 nm UV-B flux on distance from the UV-B source. UV-B flux photon data measured at various distances from the narrow band tube (symbols), and the polynomial fit (straight line and parameters) that was used for calculating photon fluxes received by plants positioned at various distances from the source.

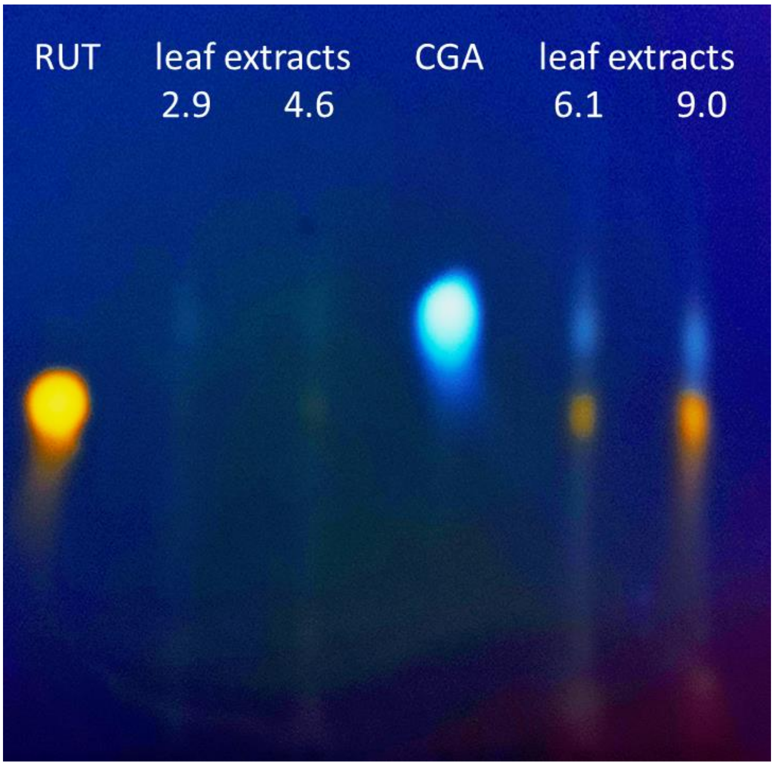

Supplementary Figure S2.

Thin layer chromatography of leaf extracts from plants exposed to various doses of 311 nm UV-B radiation. Numbers represent photon fluxes in µmol m^{-2} s^{-1} units, which were applied to the plants for 4 days, 4h daily. For reference, test compounds quercetin-3-*O*-rutinoside (RUT) and chlorogenic acid (CGA) were also applied on the TCA plate.
